# Absent pulmonary valve syndrome: Valvular reconstruction with autologous pulmonary arterial wall

**DOI:** 10.1016/j.xjtc.2025.05.010

**Published:** 2025-05-30

**Authors:** Igor E. Konstantinov, Carolina Rodrigues, Sergei I. Konstantinov, Tyson A. Fricke

**Affiliations:** a Department of Cardiothoracic Surgery, Royal Children's Hospital, Melbourne, Australia; b Department of Paediatrics, University of Melbourne, Melbourne, Australia; c Heart Research Group, Murdoch Children's Research Institute, Melbourne, Australia; d Melbourne Centre for Cardiovascular Genomics and Regenerative Medicine, Melbourne, Australia

**Figure:**
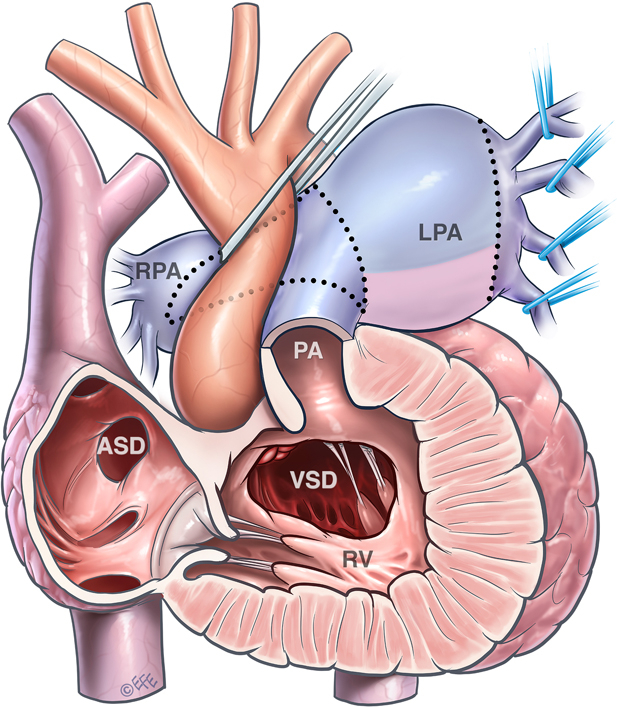
Cardiovascular anomalies in APV syndrome.

**Central Message**

APV can be reconstructed in an infant with a living autologous pulmonary arterial wall.

Tetralogy of Fallot (TOF) associated with absent pulmonary valve (APV) is a rare variant that comprises 3% to 6% of all patients with TOF.^1^ In addition to the typical intracardiac abnormalities of TOF, those with TOF/APV have a severely dysplastic pulmonary valve (PV) with free pulmonary insufficiency. The characteristic feature of APV syndrome is the aneurysmal pulmonary arteries often causing compression of the trachea and bronchi. Therefore, patients with TOF/APV often require preoperative ventilatory support and may need early neonatal surgical repair. Surgical treatment of neonates and infants with TOF/APV has been associated with increased postoperative respiratory complications and hospital mortality.^2^ Surgical strategy has been focused on reduction of the dilated pulmonary arteries, relief of compression of the tracheobronchial tree, and establishment of PV competency using valved conduits. We describe our technique of PV reconstruction using a living autologous pulmonary wall obtained from reduction of the branch pulmonary arteries. The Royal Children's Hospital Human Research Ethics Committee approved this retrospective review (HREC/21/QCHQ/80891 on November 11, 2021), and the parents of the patient provided informed written consent for the publication of the data.

A 3-month-old girl (weight 4.4 kg, height 56 cm, body surface area 0.25 m^2^) with DiGeorge syndrome was transferred from another hospital with TOF/APV. On arrival, she presented with severe stridor and required emergent intubation. Echocardiogram showed a large subaortic ventricular septal defect, severe stenosis of the right ventricular outflow tract (RVOT) with a mean pressure gradient of 64 mm Hg and free regurgitation, and large branch pulmonary arteries. Computed tomography demonstrated severe aneurysmal dilatation of the left pulmonary artery (LPA) (3.1 × 3 cm, Z score +11.2) and tubular dilatation of the right pulmonary artery (PA) (1.6 × 1.7 cm, Z score +7.2), with severe narrowing of the proximal right main bronchus and moderate narrowing of the proximal left main bronchus (Figure 1 and Video 1). The patient underwent TOF repair and reduction of the branch pulmonary arteries (Video 1). A ring of the LPA was resected, and the remainder of the LPA and the right PA were incised and plicated. The initial reconstruction of the pulmonary arteries was completed with the heart beating. After crossclamping and cardioplegic arrest, the main PA was incised across the annulus and the ring of the LPA was used to create the new PV and RVOT (Figure 2). The perimembranous ventricular septal defect was closed using a bovine pericardial patch. Aortic crossclamp time was 93 minutes, and cardiopulmonary bypass time was 256 minutes. Epicardial echocardiogram demonstrated good biventricular systolic function, no pulmonary regurgitation, and an unobstructed RVOT (Video 1). There was no pressure gradient across the PV by echocardiographic assessment or direct interoperative needle measurement.

**Figure 1:**
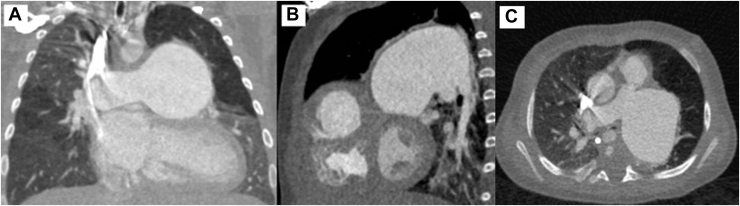
Preoperative computed tomography demonstrates aneurysmal dilatation of both branches of the PA, with severe narrowing of the right and left bronchi.

**Figure 2:**
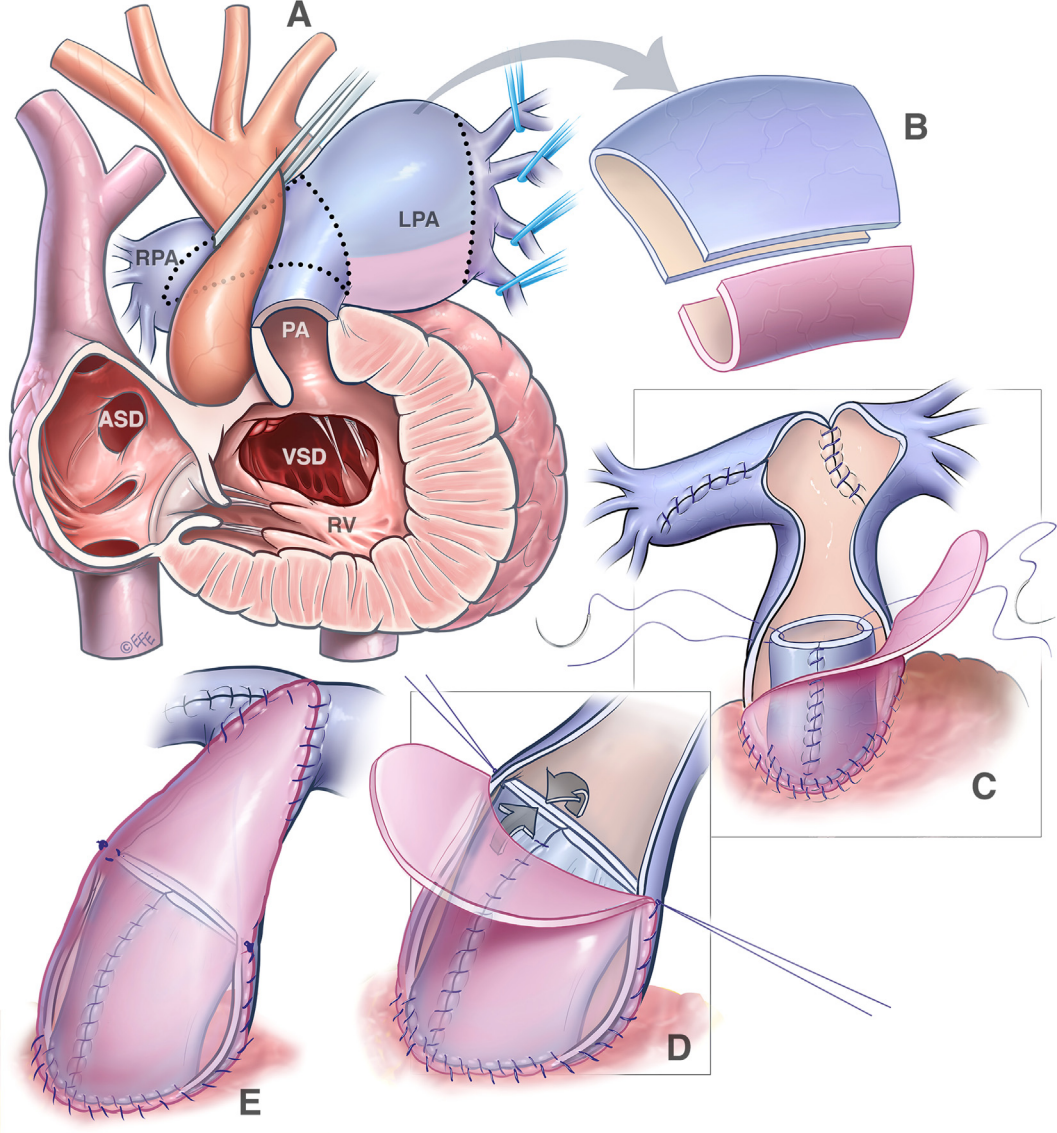
Illustration of the surgical repair. A, Enlarged branches of the PA were dissected into the hilum of each lung. B, A ring of the LPA was resected and used for reconstruction of the PV and anterior wall of the main PA. C, The LPA and right PA were reduced and reconstructed. C-E, The LPA was used to create the new PV and anterior wall of the main PA. *ASD*, Atrial septal defect; *LPA*, left pulmonary artery; *RPA*, right pulmonary artery; *RV*, right ventricle; *VSD*, ventricular septal defect.

She was extubated on postoperative day 5. The patient is asymptomatic, and the valve functions well without pressure gradient and is competent at 7 months of follow-up. The echocardiographic appearance of the reconstructed valve remains unchanged.

The patient described in this report represents the severe spectrum of the APV syndrome. Children who require preoperative mechanical ventilation and surgery early in life are at high risk of death.^2^, ^3^, ^4^ In particular, a multivariate analysis of overall survival identified that preoperative ventilation was an independent predictor for death in our previous study.^2^ Therefore, we believe that both reduction of the intra-hilar pulmonary arteries and achieving PV competence are essential for the optimal outcome.

It appears that distal airway compression plays a pivotal role in postoperative morbidity. Several maneuvers were proposed to relieve distal airway compression, including anterior and posterior plication of the dilated pulmonary arteries, reduction of the PA by excising parts of the posterior or anterior walls, suspension of the PA to the retrosternal fascia, and translocating the PA anterior to the aorta with the Lecompte maneuver.^4^, ^5^, ^6^, ^7^, ^8^, ^9^ However, these techniques may not be effective if distal airway compression persists. In our previous study, 5 of 7 operative deaths were due to persistent distal airway compression identified by postoperative bronchogram.^2^ This led us to apply the technique of intra-hilar reduction of the PA branches.^2^ We believe such reduction of pulmonary arteries in the hilum is crucial in relieving the distal bronchial compression.

It also appears that ensuring PV competence is important in these sick infants. As such, PV competence may reduce the risk of persistent distal bronchomalacia due to increased pulsatility and flow reversal in the distal pulmonary arteries, observed in those with severe PV insufficiency. It is also important for optimization of the hemodynamics. However, replacement of the PV in infancy is problematic and will require an early conduit change. Thus, alternative methods of PV reconstruction have been described.^10^ In principle, it is important to avoid a significant residual RVOT gradient. We have previously demonstrated that a postoperative RVOT obstruction peak gradient of 25 mm Hg or greater was a significant predictor for reoperation.^11^ The diameter of the reconstructed valve should be approximately equal to its height. For example, in our patient, the diameter of the RVOT was 14 mm, and the height of the new valve was about the same to ensure good coaptation. Because there is always an excess of the PA wall after resection, the technique of the PV reconstruction with living autologous tissue described appears to be an attractive option.

## Conflict of Interest Statement

The authors reported no conflicts of interest.

The *Journal* policy requires editors and reviewers to disclose conflicts of interest and to decline handling or reviewing manuscripts for which they may have a conflict of interest. The editors and reviewers of this article have no conflicts of interest.

## Supplementary Data

Video 1 The senior author explains perioperative findings and procedures. Video available at: https://www.jtcvs.org/article/S2666-2507(25)00231-7/fulltext.
