# Supplementary figures and images for: Absent pulmonary valve syndrome: Valvular reconstruction with autologous pulmonary arterial wall
Source: JTCVS Tech. 2025 May 30;32:105–7. doi: 10.1016/j.xjtc.2025.05.010 (PMC12348031; doi:10.1016/j.xjtc.2025.05.010)

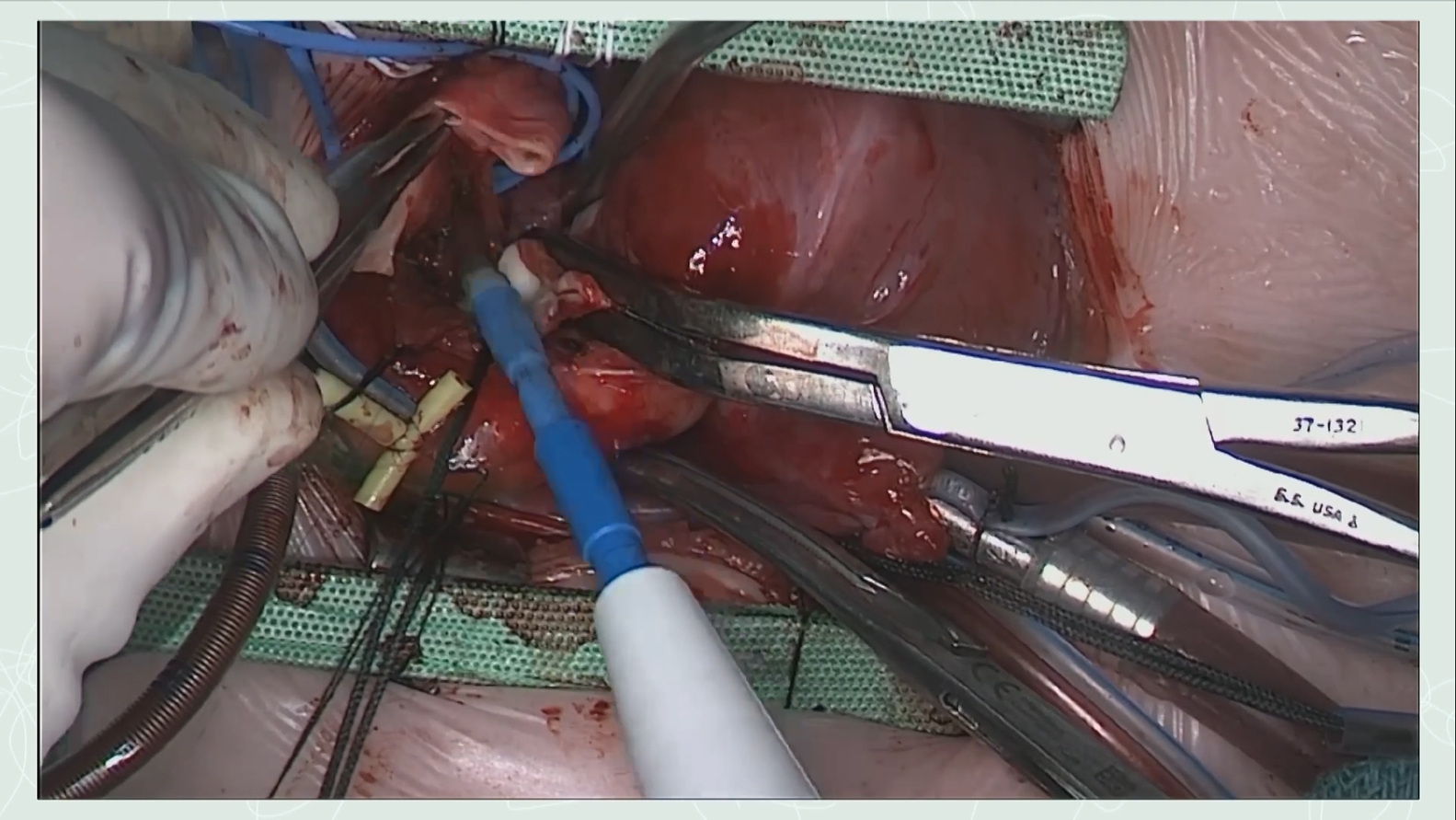

Supplement: Video 1 — The senior author explains perioperative findings and procedures. Video available at: https://www.jtcvs.org/article/S2666-2507(25)00231-7/fulltext. [file fx2.jpg]
